# Influence of the MDM2 single nucleotide polymorphism SNP309 on tumour development in BRCA1 mutation carriers

**DOI:** 10.1186/1471-2407-6-80

**Published:** 2006-03-24

**Authors:** Ellen R Copson, Helen E White, Jeremy P Blaydes, David O Robinson, Peter W Johnson, Diana M Eccles

**Affiliations:** 1 Cancer Research UK Oncology Unit, Cancer Sciences Division, University of Southampton School of Medicine, Southampton General Hospital, Southampton, SO16 6YD, UK; 2 National Genetics Reference Laboratory (Wessex), Salisbury District Hospital, Salisbury, SP2 8BJ, UK; 3 Wessex Regional Genetics Laboratory, Salisbury District Hospital, Salisbury, SP2 8BJ, UK; 4 Wessex Clinical Genetics Service, Princess Anne Hospital, Southampton, SO16 5YA, UK

## Abstract

**Background:**

The MDM2 gene encodes a negative regulator of the p53 tumour suppressor protein. A single nucleotide polymorphism (SNP) in the MDM2 promoter (a T to G exchange at nucleotide 309) has been reported to produce accelerated tumour formation in individuals with inherited p53 mutations. We have investigated the effect of the MDM2 SNP309 on clinical outcome in a cohort of patients with germline mutations of BRCA1.

**Methods:**

Genomic DNA was obtained for 102 healthy controls and 116 patients with established pathogenic mutations of BRCA1 and Pyrosequencing technology™ was used to determine the genotype at the MDM2 SNP309 locus.

**Results:**

The polymorphism was present in 52.9% of the controls (G/T in 37.3% and G/G in 15.6%) and 58.6% of the BRCA1 mutation carriers (47.4% G/T and 11.2% G/G). Incidence of malignancy in female BRCA1 carriers was not significantly higher in SNP309 carriers than in wildtype (T/T) individuals (72.7% vs. 75.6%, p = 1.00). Mean age of diagnosis of first breast cancer was 41.2 years in the SNP309 G/G genotype carriers, 38.6 years in those with the SNP309 G/T genotype and 39.0 years in wildtype subjects (p = 0.80).

**Conclusion:**

We found no evidence that the MDM2 SNP309 accelerates tumour development in carriers of known pathogenic germline mutations of BRCA1.

## Background

Inheritance of a truncating mutation of the breast cancer predisposition gene BRCA1 has been reported to carry a lifetime risk of breast cancer of between 50 and 80%, with age at onset of first malignancy varying from the 2^nd ^to the 8^th ^decade [1,2]. Clinical studies indicate that BRCA1 mutation carriers can benefit significantly from preventative surgery, with prophylactic oophorectomy reducing breast cancer rates by up to 60% and ovarian cancer rates by 95%, and prophylactic double mastectomy reducing the incidence of breast cancer by up to 90% [3,4]. These two interventions are however major surgical procedures with potentially significant psychological and medical sequelae. The ability to predict whether a BRCA1 mutation carrier is likely to develop malignant disease early or late in life would facilitate the clinical management of these patients.

No differences in penetrance have yet been documented for different BRCA1 truncating mutations [2]. Inter-individual variation in the speed at which BRCA1 mutation carriers develop cancer is likely to be influenced by environmental factors, but may also be affected by co-inheritance of other disease modifying genes which interact with the BRCA1 cellular pathway. The precise mechanisms by which mutations in the BRCA1 gene result specifically in breast and ovarian tumour growth remain to be elucidated. It is however accepted that BRCA1, and BRCA2, maintain genomic integrity through roles in transcriptional regulation, homologous recombination and the DNA damage response [5-7] and are required for the normal proliferative burst in early embryogenesis [8,9].

It has been noted that BRCA1 breast tumours have a higher frequency of p53 mutations than sporadic breast tumours and several lines of evidence now point to a role for the tumour suppressor p53 in the development of BRCA1 cancers [10-14]. Mice carrying homozygous mutations of BRCA1 fail to develop beyond an early embryonic stage but have their survival prolonged by the co-inheritance of p53 mutations [15,16]. tBRCA1-/- mice carry a targeted null mutation of BRCA1 restricted to the T-cell lineage resulting in depletion of thymocytes and peripheral T-cells, and activation of p53 [17]. Thymocyte development in these mice is restored in the absence of p53. Such observations have led to the hypothesis that truncating mutations in BRCA1 lead to accumulation of DNA damage and activation of cell cycle checkpoints including activation of p53, with the subsequent suggestion that disruption of the p53 pathway may be required before a BRCA1 mutation results in tumour development [7].

The co-inheritance of alterations in the p53 tumour suppressor pathway would therefore be expected to modify the clinical affects of a BRCA1 mutation. Pre-clinical studies by McPherson *et al*. have provided evidence of the role Chk2 plays in mediating accumulation and activation of p53 in BRCA1 deficient mice [18]. A deficiency of Chk2 partially rescued the defective development and growth of BRCA1 deficient T-cells but enhanced tumour development. Recently Bond *et al*, (2004) have reported a single nucleotide polymorphism (SNP) within the MDM2 gene that can result in a partially attenuated p53 response and accelerated tumour development in carriers [19].

MDM2 encodes a protein which directly binds to and inhibits p53 [20] but also has p53 independent negative effects on DNA double strand break repair [21,22]. MDM2 gene amplification is found in a number of tumour types, including sporadic breast cancers, [23] and overexpression of MDM2 is associated with accelerated tumour development and failure to respond to treatment [24]. Bond *et al*, (2004) have described a T to G exchange at nucleotide 309 within the promoter region of MDM2, which increases the affinity of the transcriptional activator Sp1 and results in higher levels of MDM2 protein with subsequent attenuation of the p53 tumour suppression pathway [19]. Their study of 88 individuals with inherited p53 mutations demonstrated that carriers of this SNP in either its' homozygous or heterozygous form developed cancer on average 7 years before their wildtype counterparts. Additionally, individuals homozygous for SNP309 but with no known hereditary cancer predisposition showed a significantly earlier age of onset of sporadic soft tissue sarcoma than those with the T/T genotype.

Given the evidence for interaction between the p53 stress response and the BRCA1 cellular pathway, MDM2 could be a plausible disease modifying gene in BRCA1 mutation carriers. We have therefore investigated the effect of the MDM2 SNP309 on clinical outcome in a cohort of patients with germline mutations of BRCA1 with established pathogenic potential.

## Methods

### Subjects

Potential study participants with previously diagnosed truncating mutations of BRCA1 were identified from the database of the Wessex Clinical Genetics Service (WCGS), Southampton. Genomic DNA was obtained for 116 BRCA1 mutation carriers, 20 male and 96 female. Age at time of screening for BRCA1 mutations ranged between 29 and 57 years. The clinical histories of all subjects were reviewed to ascertain age at first and subsequent malignancies. Fifty-nine gene carriers had developed breast cancer and a further 14 had ovarian cancer. Ten patients had undergone prophylactic bilateral oophorectomy (five after developing breast cancer) and 3 patients had undergone bilateral risk reducing mastectomy. All malignancies occurred in female subjects; male subjects were therefore excluded from all analyses of cancer incidence. 102 anonymous genomic DNA samples, (46 male, 56 female, age range 16–82) referred by WCGS for genetic screening of non-neoplastic conditions, were obtained to provide an unmatched control group. All research was performed with the approval of the South West Hampshire Local Research Ethics Committee (study reference numbers: 05/Q1702/154 and MREC98/5/27).

### Genotyping

Genotyping was performed using Pyrosequencing™ technology. Amplicons were generated in a 50 μl reaction volume with 15 pmol each of MDM2F (5' GTCTCCGCGGGAGTTCA 3') and MDM2R (5' Biotin – GACTACGCGCAGCGT TCAC 3'), 0.2 mM dNTPs, 1.5 mM MgCl_2_, 1X Buffer II (Applied Biosystems), 1U AmpliTaq Gold (Applied Biosystems) using 10 ng genomic DNA. PCR conditions were 94°C for 7 min; 50 cycles with denaturation at 94°C for 30s, annealing at 60°C for 30s and elongation at 72°C for 30s; 1 cycle at 72°C for 7 min; and a final hold at 15°C. Thermocycling was performed using a PTC-0225 DNA Engine Tetrad (MJ Research).

Single-stranded biotinylated PCR products were prepared for Pyrosequencing using a Vacuum Prep Tool (Biotage AB). 3 μl Streptavidin Sepharose™ HP (Amersham) was added to 37 μl Binding buffer (10 mM Tris-HCl pH 7.6, 2 M NaCl, 1 mM EDTA, 0.1% Tween 20) and mixed with 20 μl PCR product and 20 μl high purity water for 10 min at room temperature using a Variomag Monoshaker (Camlab). The beads containing the immobilised templates were captured onto the filter probes after applying the vacuum and then washed with 70% ethanol for 5 sec, denaturation solution (0.2 M NaOH) for 5 sec and washing buffer (10 mM Tris-Acetate pH 7.6) for 5 sec. The vacuum was switched off and the beads released into a PSQ 96 well plate containing 45 μl annealing buffer (20 mM Tris-Acetate, 2 mM MgAc_2 _pH 7.6), 0.3 μM MDM2 sequencing primer (5'GGGCTGCGGGGCCGCT 3'). The samples were heated to 80°C for 2 min and then allowed to cool to room temperature.

Pyrosequencing reactions were performed according to the manufacturer's instructions using the PSQ 96 SNP Reagent Kit (Biotage AB) which contained the enzyme and substrate mixture and nucleotides. Assays were performed using the nucleotide dispensation order AGTACGCGC. The sample genotype was determined using SNP Software (Biotage AB).

### Statistical Analysis

Comparisons of frequencies were analysed using Fisher's Exact for 3 × 2 contingency tables. A one way ANOVA was used to compare age of onset of malignancies. All statistical calculations were performed using StatXact™ version 5 or Minitab™ statistical analysis software as appropriate.

## Results

The heterozygous form of SNP309 (G/T) was present in 38 of the 102 controls (37.3%) with the homozygous form present in 16 (15.6%). This distribution was not significantly different from that within the BRCA1 mutation carriers: 13 /116 (11.2%) were G/G, 55 /116 (47.4%) were G/T and 48/116 (41.4%) were T/T, (p = 0.286).

Seventy-one of the 96 female BRCA1 mutation carriers had been diagnosed with at least one malignancy of any histological type (table 1). The incidence of malignancy was highest in G/G genotype carriers with these patients developing tumours in 10/12 cases (83.3.%). G/T carriers developed tumours in 30/43 cases (69.8%) and wildtype (T/T) individuals developed tumours in 31/41 cases (75.6%), but statistical analysis revealed no significant association between incidence of malignancy and genotype (p = 0.61).

**Figure 1:**
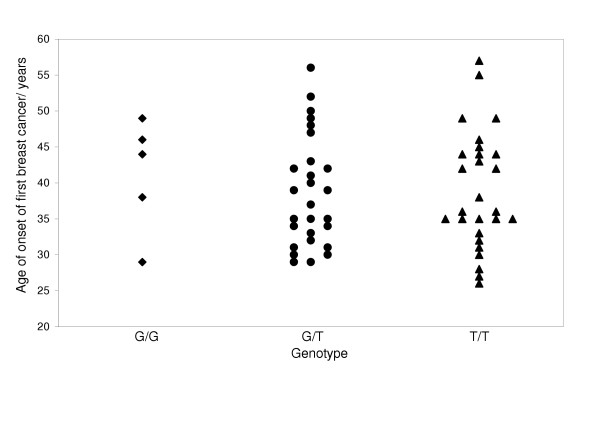
Scatter plot of age of onset of first breast cancer in carriers of pathogenic BRCA1 mutations genotyped for MDM2 SNP309.

Breast cancer had developed in 59 of the females with BRCA1 mutations: 5/12 (41.7%) of those with the G/G genotype, 27/43 (62.8%) of those with the G/T genotype and 27/ 41 (65.8%) of those with the T/T genotype. Mean age of diagnosis of first breast cancer did not vary significantly with genotype, being 41.2 years (range 29–49) in the SNP309 G/G carriers, 38.6 years (range 29–52) in SNP G/T carriers, and 39.0 years (range 26–57) in subjects with the wildtype genotype (p = 0.80) (figure 1).

Ovarian cancers occurred in 14 females: 4 with G/G genotype (33.3%), 4 with G/T (9.3%) and 6 with T/T (14.6%, p = 0.32). Mean age of onset was similar in each genotype group being 62.3 years in the G/G group (range 51–74), 60.3 years in the G/T group (range 51–68 years) and 53.7 years in the wildtype group (range 44–66), p = 0.32.

## Discussion

Analysis of our control group of 102 confirmed that the MDM2 SNP309 genetic variation is relatively common within the general population with a frequency of 37.3% for the heterozygous state (G/T) and 15.6% for the homozygous state (G/G). Our figure for the homozygous frequency is slightly higher than that reported by Bond *et al*, (2004) in their analysis of 50 healthy volunteers (40% G/T, 12% G/G) but is similar to that found by Campbell *et al*. in another control group from the UK [19,25]. We have shown that distribution of this polymorphism within carriers of pathogenic BRCA1 mutations is not significantly different from that within the general population.

Our group of 116 BRCA1 mutation carriers had developed a significant number of malignancies (total incidence 61.2%) but our work has shown no evidence of a significant association between genotype at MDM2 nucleotide 309 and incidence of breast or ovarian cancer in female subjects. There is however a non-significant trend for increased incidence of all malignancies and of ovarian cancer in the G/G genotype group. The incidence of malignancy is so high in each group that it would be difficult to observe a statistically significant difference between subgroups in this size study population. The number of subjects in this study was limited by availability of BRCA1 mutation positive DNA within the Wessex Clinical Genetics Service; extension of this study to additional regional genetics services would increase its statistical power. A recent study by Campbell et al. found no significant association between the G/G genotype and breast cancer in a group of 218 early onset breast cancer patients (age at onset of less than 40 years) or in a group of 248 patients with familial breast cancer (defined as two or more cases of breast cancer reported in first or second-degree relatives or bilateral breast cancer) [25]. The BRCA1 mutation status of Campbell's subjects was however not reported.

It should be noted that the mean age at time of BRCA1 testing for the G/G patients is significantly higher than that of the G/T and T/T groups at 58.7 years, compared with 49.9 years for G/T carriers and 48.9 years for wildtype patients (p = 0.034). The G/G group therefore have had longer to develop malignancies than the other subjects. In addition, 5 patients in each of the G/T and T/T groups had undergone prophylactic oophorectomy whilst no patients with the G/G genotype had had this procedure. This may have affected the incidence of ovarian cancers in the G/T and T/T groups and could also have reduced the frequency of breast cancers in the 5 subjects (4 G/T, 1 T/T) who had not already developed this disease. The trend for increased incidence of ovarian cancers in the G/G group does perhaps warrant further study in a larger cohort of patients who have not undergone prophylactic surgery.

Of most interest is the fact that we found no association between SNP309 genotype and age of onset of breast cancer in female subjects with pathogenic BRCA1 mutations. Pre-clinical studies have shown clear evidence of an interaction between the p53 tumour suppressor and BRCA1 cellular pathways. It could therefore be expected that partial attenuation of the p53 pathway associated with the MDM2 SNP309, as described by Bond *et al*. would have exacerbated the effect of the BRCA1 mutation in promoting early onset breast cancer [19]. Our results may indicate that the increased MDM2 levels associated with the SNP309 are insufficient to attenuate response in the BRCA1 mutant breast cells, whereas in the Li Fraumeni cohort studied by Bond *et al*, there is already loss of one p53 allele. Alternatively, the transcription factors influenced by SNP309 could be cell type specific so that this polymorphism does not affect MDM2 expression in breast and ovarian tissue. MDM2 amplification has in fact been reported to be rare in ovarian cancers regardless of p53 mutation status, suggesting that this gene may not be significantly involved in the development of these tumours [26]. Overexpression of MDM2 has however been found in up to 73% of breast cancers [23] and breast cancers comprised 17 out of 66 of the tumours reported by Bond's cohort of Li Fraumeni individuals [19].

P53 mutations are the most commonly found genetic alteration in human tumours and a number of studies have reported a higher frequency of p53 mutations in BRCA1 associated breast tumours and ovarian cancers than sporadic breast/ ovarian cancers [11,13,15,27]. The p53 status of breast cancers occurring in our subjects is however currently unknown and it is therefore possible that an association between accelerated tumour development and MDM2 SNP309 would be found if analyses were restricted to BRCA1 mutation carriers with evidence of associated p53 protein abnormalities in the breast tumour.

Our results are consistent with those of Campbell *et al*. who found no evidence that the G/G genotype accelerated breast cancer development in 147 patients with a positive family history of this malignancy or bilateral breast cancer, a proportion of whom are likely to carry BRCA1 mutations [25]. Bond *et al*, (2004) found clear evidence of accelerated tumour development in 88 individuals carrying germline mutations of p53 and in 105 soft tissue sarcoma patients with no known familial tendency. More recently, this group have commented on the disparity in the difference in mean age of disease onset between the G/G and T/T groups of sporadic sarcomas (12 years) and the difference in median age of disease onset (21 years). These figures arise as a result of a few G/G individuals developing cancer at a later age comparable with the wildtype individuals. Bond *et al*, have attributed these findings to the existence of additional genetic modifiers [28]. Alhopuro *et al*, have now published a study in which no statistically significant association between SNP309 genotype and patient age at disease onset was found in cohorts of early uterine leiomyosarcoma (68 subjects), colorectal cancer (1042 subjects) or squamous cell head and neck cancer (162 subjects) [29]. These results together with our own findings suggest that the influence of the MDM2 SNP309 on tumour development is specific to certain patient subgroups.

## Conclusion

We have found no evidence that the presence of the MDM2 SNP 309 accelerates tumour development in carriers of known pathogenic germline mutations of BRCA1. This polymorphism seems unlikely to act as a significant disease modifier in this population.

## Competing interests

The author(s) declare that they have no competing interests.

## Authors' contributions

ERC carried out the molecular genetic studies, performed the statistical analysis and drafted the manuscript, HEW designed and optimised the pyrosequencer assay, DOR participated in the design of the study and supervised the laboratory work, JPB, PWJ and DME conceived of the study, and participated in its design and coordination. All authors read and approved the final manuscript.

## Pre-publication history

The pre-publication history for this paper can be accessed here:



## Figures and Tables

**Table 1:** Incidence and age of onset of all malignancies, breast and ovarian cancer in carriers of pathogenic BRCA1 mutations genotyped for MDM2 SNP309 (p values refer to comparisons of frequencies between the G/G, G/T and T/T subgroups)

**Genotype (female subjects only)**	**Total ** n = 96	**G/G ** n = 12	**G/T ** n = 43	**T/T ** n = 41	
Number of subjects with ≥1 malignancy (%)	71 (74.0%)	10 (83.3%)	30 (69.8%)	31 (75.6%)	p = 0.61
No. of subjects with breast cancer (%)	59 (61.5%)	5 (41.7%)	27 (62.8%)	27 (65.8%)	p = 0.34
Mean age of onset of 1^st ^breast cancer/years (range)	41.6 (26–57)	41.2 (29–49)	38.6 (29–52)	39.0 (26–57)	p = 0.80
No. of subjects with ovarian cancer (%)	14 (14.6%)	4 (33.3%)	4 (9.3%)	6 (14.6%)	p = 0.32
Mean age of onset of 1^st ^ovarian cancer/years (range)	58.0 (44–74)	62.3 (51–74)	60.3 (51–68)	53.7 (44–66)	p = 0.32
Other malignancies	3	1 plasmacytoma	1 cervical cancer 1 uterine cancer		
No. of patients undergoing prophylactic bilateral oophorectomy	10	0	5	5	
No. of patients undergoing bilateral risk reducing mastectomy	3	1	1	1	
